# Suppression of salt-induced aggregation of 4-mercaptobenzoic acid functionalized gold nanoparticles by aromatic dye molecules

**DOI:** 10.1039/d6ra06586d

**Published:** 2026-09-23

**Authors:** Kazi Mohammed Mehedi Hassan, Towfiq Ahmed Chowdhury, Monika Tarafdar, Md. Anisuzzaman, Mostafizur Rahaman, Mohammad Anwar Parvez, Jamal Uddin, Mohammad A. Hasnat, Md. Saiful Alam

**Affiliations:** a Department of Chemistry, School of Physical Sciences, Shahjalal University of Science and Technology Sylhet 3114 Bangladesh saifulalam-che@sust.edu; b Pharmacy Discipline, Khulna University Khulna 9208 Bangladesh; c Department of Chemistry, College of Science, King Saud University P.O. Box 2455 Riyadh 11451 Saudi Arabia; d Department of Chemical Engineering, College of Engineering, King Faisal University P.O. Box 380 Al-Ahsa 31982 Saudi Arabia; e Center for Nanotechnology, Department of Natural Sciences, Coppin State University 2500 W. North Ave. Baltimore MD 21216 USA; f Electrochemistry & Catalysis Research Laboratory (ECRL), Department of Chemistry, School of Physical Sciences, Shahjalal University of Science and Technology Sylhet 3114 Bangladesh

## Abstract

The colloidal instability of ligand-functionalized gold nanoparticles under electrolyte-rich conditions limits their application in plasmonic sensing. Here, 4-mercaptobenzoic acid-functionalized gold nanoparticles (Au–4MBA) were investigated for their aggregation behavior in NaCl-containing media and stabilization by aromatic dyes. Au–4MBA nanoparticles exhibited an LSPR band at 524 nm, while exposure to 5 mM NaCl induced aggregation, characterized by a secondary plasmon band at ∼670 nm and a visible color change. Congo Red (CR) progressively suppressed this aggregation-associated response with increasing concentration. The colloidal stability was strongly pH-dependent but remained relatively insensitive to temperature over 10–50 °C. Comparative evaluation of structurally distinct dyes revealed different stabilization behaviors, following the order CR ≈ Erythrosine B > Methyl Orange > Alizarin Red S >> Methylene Blue. These differences are consistent with a structure-dependent influence of aromatic dyes on nanoparticle stability. The experimental observations support a proposed electrosteric stabilization mechanism involving possible interfacial association and noncovalent interactions that restrict close nanoparticle approach and plasmonic coupling. These findings provide a simple strategy for improving the electrolyte stability of ligand-functionalized plasmonic nanoparticles.

## Abstract 2

The colloidal instability of ligand-functionalized gold nanoparticles under electrolyte-rich conditions limits their application in plasmonic sensing.

## Graphical abstract

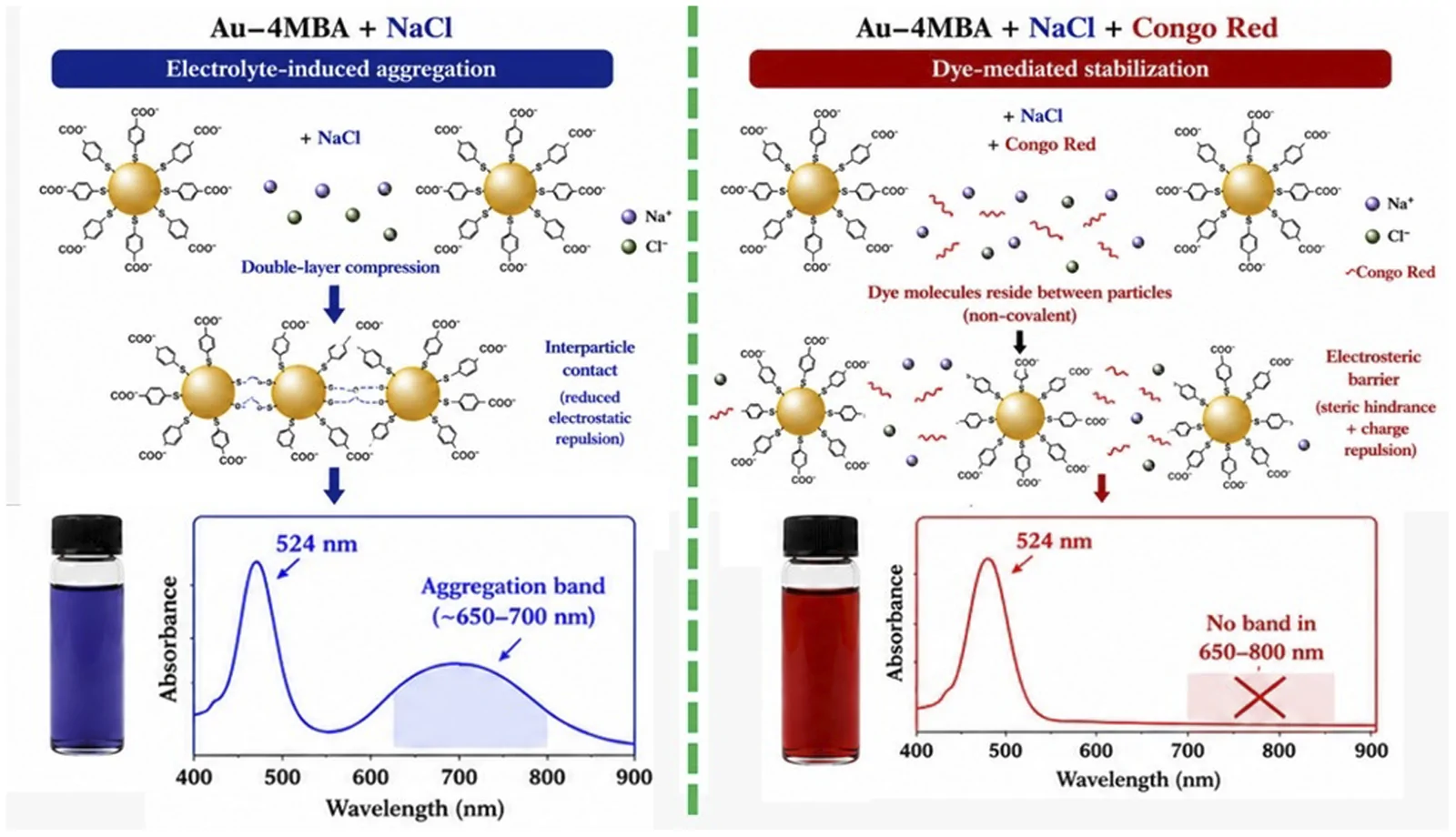

## 1. Introduction

Gold nanoparticles (AuNPs) are among the most widely studied plasmonic nanomaterials owing to their unique localized surface plasmon resonance (LSPR), which is highly sensitive to particle size, morphology, interparticle distance, and the surrounding dielectric environment.^1–3^ Based on these properties, Au NPs has numerous applications particularly in colorimetric sensing, surface-enhanced Raman scattering (SERS), bioanalysis, catalysis, and environmental monitoring.^4–6^ However, the interface of nanoparticles is significant for determining the optical and colloidal responses. Therefore, surface ligands have a critical role in the interaction of external molecular species with the nanoparticles, which has influences on their assembly behaviour.^7^ Moreover, the colloidal stability depends on the ligands structure, ionization state, or molecular adsorption at the nanoparticle interface. Interfacial ligand interactions govern aggregation and stabilization of nanoparticles that can be ensured by the surface functionalization of nanoparticles. Surface functionalization is a crucial strategy for tailoring the physicochemical properties of gold nanoparticles (AuNPs). Functional ligands can regulate surface charge, colloidal stability, molecular recognition, and interparticle interactions, thereby influencing both the optical response and aggregation behaviour of AuNPs.^8–10^ In addition, surface functionalization introduces specific chemical functionalities that enable targeted sensing, selective adsorption, and controlled assembly of nanoparticles.

Among the various ligand-functionalized AuNP systems, 4-mercaptobenzoic acid (4MBA)-modified nanoparticles have attracted considerable attention due to containing a gold-binding thiol group and a terminal carboxylic acid functionality that provides a chemically accessible interface.^7,11,12^ The strong Au–S interaction ensures stable surface attachment and the exposed carboxyl group can contribute to surface charge regulation and colloidal stability. As a result, Au–4MBA nanoparticles have been extensively employed in plasmonic sensing and SERS-based applications.^11–13^ The optical properties of AuNPs are strongly dependent on their aggregation state. Nanoparticles exhibit a characteristic LSPR band under the dispersed conditions, whereas interparticle association results in plasmonic coupling, spectral broadening, and visible colour changes.^14–16^ In ligand-functionalized systems, aggregation is commonly induced by electrolytes, which compress the electrical double layer and reduce electrostatic repulsion between neighbouring particles.^17,18^ Aggregation-induced optical changes are widely used as sensing signals.^19^ However, more research has mainly focused on molecules that promote nanoparticle aggregation. Less attention has been paid to molecules that inhibit aggregation and maintain colloidal stability under electrolyte conditions. This is especially relevant for ligand-functionalized nanoparticles, where molecular adsorption can alter surface charge and interparticle interactions, thereby influencing colloidal stability and aggregation behaviour. Aromatic dye molecules provide a useful model for investigating these effects because they possess diverse molecular architectures, charge distributions, and functional groups capable of interacting with nanoparticle interfaces.^20,21^ Their extended π-conjugated frameworks and charged substituents can influence adsorption behaviour, interfacial organization, and colloidal stability.^22–24^ However, unlike previous studies that primarily focused on the synthesis of functionalized gold nanoparticles, the adsorption characteristics of individual thiol ligands, or the use of nanoparticle aggregation as a sensing mechanism, the present work establishes a systematic structure–interaction–stability relationship for aromatic dye-mediated stabilization of Au–4MBA nanoparticles under electrolyte-rich conditions. Additionally, limited studies have explored how differences in dye structure influence the stabilization of ligand-functionalized AuNPs under electrolyte conditions have been investigated herein.

In this work, citrate-stabilized AuNPs were functionalized with 4MBA to generate Au–4MBA nanoparticles, and their aggregation behaviour was systematically investigated under electrolyte conditions. We hypothesized that adsorption of aromatic dye molecules onto the Au–4MBA interface could modify interparticle interactions and suppress electrolyte-induced aggregation through electrostatic and steric stabilization effects. To examine this possibility, the influence of ionic strength, dye concentration, incubation time, pH, and temperature on colloidal stability was evaluated. Furthermore, a comparative study involving CR, EB, ARS, MO, and MB was conducted to establish a structure–stability relationship governing dye-mediated stabilization. The findings provide a mechanistic perspective on aggregation suppression in ligand-functionalized plasmonic nanoparticles and offer a framework for designing more robust plasmonic systems capable of operating under electrolyte-rich conditions.

## 2. Materials and methods

### 2.1. Chemicals and reagents

Hydrogen tetrachloroaurate(III) trihydrate (HAuCl_4_·3H_2_O), trisodium citrate dihydrate, 4-mercaptobenzoic acid (4-MBA), Congo Red (CR), sodium chloride (NaCl), sodium hydroxide (NaOH), Dichloromethane (DCM) and hydrochloric acid (HCl) were purchased from commercial suppliers and used without further purification. Ultrapure water (resistivity of 18.2 MΩ cm) was used in all experiments. Glassware used for nanoparticle synthesis was thoroughly cleaned with aqua regia, rinsed several times with ultrapure water, and dried prior to use.

### 2.2. Instrumentation

UV–visible absorption spectra were recorded using a double-beam UV–visible spectrophotometer equipped with a 1 cm quartz cuvette over the wavelength range of 200–800 nm. The morphology and particle size of the synthesized nanoparticles were examined by transmission electron microscopy (TEM) operated at an accelerating voltage of 200 kV. Samples for TEM analysis was prepared by drop-casting dilute nanoparticle suspensions onto carbon-coated copper grids and drying under ambient conditions. SERS spectra were collected using a confocal Raman microscope equipped with a 785 nm diode laser, a 50× objective lens, and a thermoelectrically cooled CCD detector. Spectra were acquired over the Raman shift range of 400–2000 cm^−1^ with an integration time of 5–10 s and typically averaged over 3 accumulations. The spectrometer was calibrated using the silicon band at 520.7 cm^−1^ prior to data acquisition.

### 2.3. Synthesis of citrate-stabilized Au nanoparticles

Citrate-stabilized Au nanoparticles were synthesized according to the Turkevich method with slight modification.^8^ Briefly, 100 mL of aqueous HAuCl_4_ solution (1.0 mM) was heated to reflux under vigorous magnetic stirring. Once boiling was established, 10 mL of trisodium citrate solution (38.8 mM) was rapidly added to the gold precursor solution. The reaction mixture was maintained under reflux for 15 min, during which the solution color gradually changed from pale yellow to colorless, then dark grey, and finally wine red, indicating the formation of colloidal gold nanoparticles. The reaction vessel was then removed from the heat source and allowed to cool naturally to room temperature under continuous stirring. The resulting colloidal suspension was stored at 4 °C and used for further surface modification.

### 2.4. Surface modification of Au nanoparticles with 4-mercaptobenzoic acid

Au–4MBA nanoparticles were prepared through ligand exchange between citrate-stabilized Au nanoparticles and 4-mercaptobenzoic acid. A freshly prepared stock solution of 4-MBA was prepared in DCM and mixed with the citrate-stabilized Au nanoparticle suspension under gentle stirring. In a typical modification procedure, 1 mL of 4-MBA stock solution (10 mM) was added to 1 mL of the citrate-stabilized Au nanoparticle dispersion, and the mixture was stirred at room temperature for 30 min to allow sufficient ligand exchange to obtain Au-4MBA NPs.^20,21^

### 2.5. Salt-induced aggregation of Au–4MBA nanoparticles

To investigate the salt sensitivity of the Au–4MBA nanoparticles, NaCl solutions of different concentrations were prepared in ultrapure water. In a typical experiment, an appropriate volume of NaCl stock solution was added to a fixed volume of Au–4MBA suspension to obtain final NaCl concentrations in the range of 1–10 mM. After mixing, the solutions were gently vortexed or stirred and allowed to equilibrate for 5–10 min prior to UV–visible measurement.

### 2.6. Spectral correction for intrinsic dye absorption

To evaluate the contribution of the intrinsic absorption of CR to the UV–visible spectra of the Au–4MBA/NaCl/CR system, control spectra of CR were recorded at concentrations corresponding to those used in the nanoparticle stabilization experiments (1, 5, 10, 20, and 50 µM). Concentration-matched CR solutions containing 5 mM NaCl were also measured under identical experimental conditions. The contribution of CR absorption to the composite spectra was evaluated using the corresponding CR/NaCl control spectrum. Corrected nanoparticle spectra were obtained according to*A*_corr_(*λ*) = *A*_Au–4MBA+NaCl+CR_(*λ*) − *A*_NaCl+CR_(*λ*)where, *A*_corr_ represents the corrected absorbance, *A*_Au–4MBA+NaCl+CR_ is the experimentally measured composite (with particle) spectrum, and *A*_NaCl+CR_ is the concentration-matched dye control spectrum.

For the comparative dye study, the intrinsic absorption of ARS, CR, EB, MO, and MB was similarly considered by recording the corresponding dye controls under the same solution conditions used for the nanoparticle experiments. Each control spectrum was used as the background for its respective Au–4MBA/NaCl/dye spectrum.

### 2.7. Determination of free and Au-4MBA aassociated CR

To evaluate the association of CR with Au–4MBA nanoparticles, Au–4MBA dispersions containing 5 mM NaCl were incubated with CR at concentrations of 1, 5, 10, 20, and 50 µM under the same conditions used for the concentration-dependent stabilization experiments. Following incubation, the nanoparticle dispersions were centrifuged to separate the nanoparticle-containing fraction from the supernatant. The supernatants were carefully collected, and their UV–Vis absorption spectra were recorded. A control containing Au–4MBA and 5 mM NaCl without CR was subjected to the same centrifugation procedure to evaluate any residual nanoparticle contribution to the supernatant. The control supernatant exhibited negligible absorbance in the visible region, indicating effective separation of the nanoparticles.

The concentration of free CR remaining in each supernatant (*C*_free_) was determined using a calibration curve constructed from CR standards under the corresponding electrolyte conditions. The concentration of CR associated with the nanoparticle-containing fraction was estimated according to:*C*_supernatant_ ≈ *C*_free_*C*_associated_ = *C*_0_ − *C*_free_where *C*_0_ represents the initial CR concentration. The percentage association was subsequently calculated as:
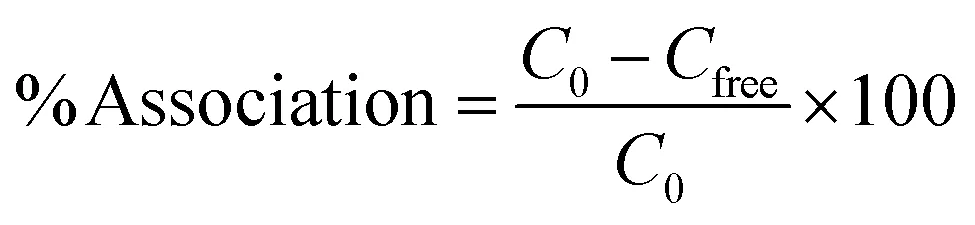


### 2.8. Aggregation index and statistical analysis

The extent of nanoparticle aggregation was quantitatively evaluated using the aggregation index (AI), calculated from the UV–visible absorbance ratio according to:
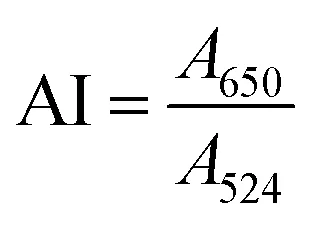
where *A*_650_ and *A*_524_ represent the absorbance at 650 nm and the characteristic LSPR wavelength of Au–4MBA nanoparticles at 524 nm, respectively. For statistical evaluation, the experiments were performed in three independent replicates (*n* = 3), and AI was calculated separately for each replicate. The results are reported as mean ± standard deviation (SD), with error bars representing the SD of the three independent measurements.

## 3. Result and discussion

### 3.1. Synthesis and characterization of Au–4MBA nanoparticles

Citrate-AuNPs were synthesized using the Turkevich reduction method and subsequently employed as the precursor for surface functionalization with 4MBA. TEM revealed the formation of well-dispersed spherical nanoparticles with no detectable aggregation (Fig. 1A).

**Fig. 1:**
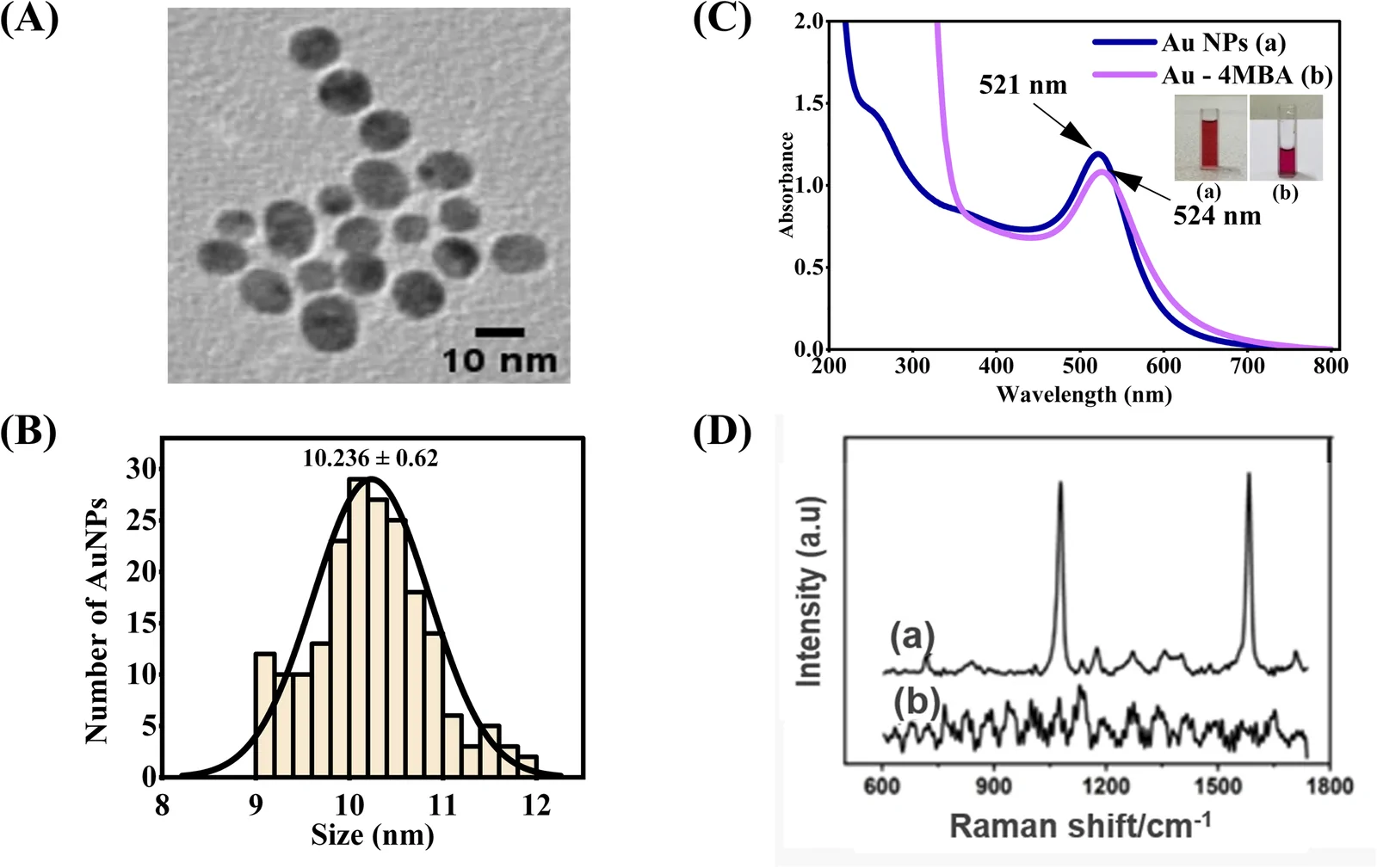
Characterization of citrate-stabilized gold nanoparticles (AuNPs). (A) TEM image showing the morphology and dispersion of the synthesized AuNPs. (B) Particle size distribution histogram obtained from TEM analysis, indicating an average particle diameter of approximately 10 nm. (C) UV–visible absorption spectrum of the AuNP colloidal suspension showing a band at 521 nm. Insets show the corresponding photographic image of the colloidal dispersion. (D) SERS spectrum of 4MBA modified Au NPs (a) and bare Au NPs (b).

Particle-size analysis showed that the average size of the synthesized particles was 10 nm with a narrow size distribution (Fig. 1B), confirming the formation of a relatively monodisperse colloidal suspension. The corresponding UV–visible spectrum exhibited LSPR band at ∼521 nm together with the characteristic wine-red appearance of the dispersion (Fig. 1C). The narrow plasmon band and absence of long-wavelength absorption are consistent with dispersed spherical AuNPs and indicate good colloidal stability following synthesis.

Surface functionalization was achieved through ligand exchange between citrate and 4-MBA, driven by the strong affinity of the thiol group toward the gold surface. The evidence for ligand functionalization was obtained from UV–visible spectroscopy (Fig. 1C). Due to functionalization, the LSPR band shifted from 521 to 524 nm, corresponding to a red shift of approximately 3 nm. This shift is commonly associated with modification of the local dielectric environment surrounding the nanoparticle surface following adsorption of an organic ligand layer.^27,28^ The formation of the Au–4MBA interface was further confirmed by SERS spectroscopy (Fig. 1D). In contrast to citrate-capped AuNPs, which produced no discernible Raman features under identical conditions whereas the functionalized nanoparticles exhibited two intense bands centered at approximately 1075 and 1587 cm^−1^. The band at 1075 cm^−1^ is assigned to the aromatic ring breathing mode coupled with C–S stretching, while the feature at 1587 cm^−1^ originates from aromatic C=C stretching vibrations of the 4-MBA molecule.^25,26^ The appearance of these characteristic bands confirms the successful immobilization of 4MBA on the nanoparticle surface through Au–S bond formation.

### 3.2. Salt-induced aggregation behaviour of Au–4MBA nanoparticles

The influence of ionic strength on the colloidal stability of Au–4MBA nanoparticles was systematically investigated by monitoring their UV–visible absorption spectra following the addition of NaCl at concentrations ranging from 1 to 10 mM (Fig. 2A). At NaCl concentrations between 1 and 4 mM, the LSPR band remained essentially unchanged, with the peak position maintained at 524 ± 1 nm and no observable long-wavelength absorption feature. The narrow and symmetric spectral profile indicates preservation of the dispersed colloidal state despite the increasing ionic strength. The corresponding photographic images further confirm this observation, as the suspensions retained their characteristic wine-red colour without visible precipitation or turbidity. Under these conditions, the negatively charged carboxylate groups on the 4-MBA-coated nanoparticle surface generate sufficient repulsion between neighbouring particles, preventing close approach and maintaining colloidal stability.^29,30^ When the NaCl concentration was increased to 5 mM, clear spectral changes became evident. A new spectral feature becomes visible in the 600–700 nm region where the primary plasmonic band was remained constant at ∼524 nm. The color of Au-4MBA colloidal solution turned from wine-red to bluish-purple supports the appearance of second plasmonic band indicating the onset of Au-4MBA nanoparticle aggregation. The emergence of this long-wavelength band indicates that the Au–4MBA nanoparticles are moving closer together due to electrolyte-induced destabilization. The reduced interparticle distance allows plasmonic coupling between neighbouring nanoparticles, resulting in the observed spectral feature. A further increase in NaCl concentration from 6 to 10 mM produced a progressive increase in absorption within the 680–700 nm region, together with a gradual decrease in the relative intensity of the primary LSPR band, demonstrating an increasing degree of nanoparticle aggregation. At 7 mM NaCl, the aggregation-associated band reached an intensity comparable to that of the primary LSPR peak, indicating that aggregation had become a leading feature of the colloidal system. Further increasing the NaCl concentration to 8–10 mM resulted in a strong long-wavelength absorption feature that dominated the spectra. Consistent with this behaviour, the suspensions exhibited a pronounced blue colour, revealing extensive aggregation.

**Fig. 2:**
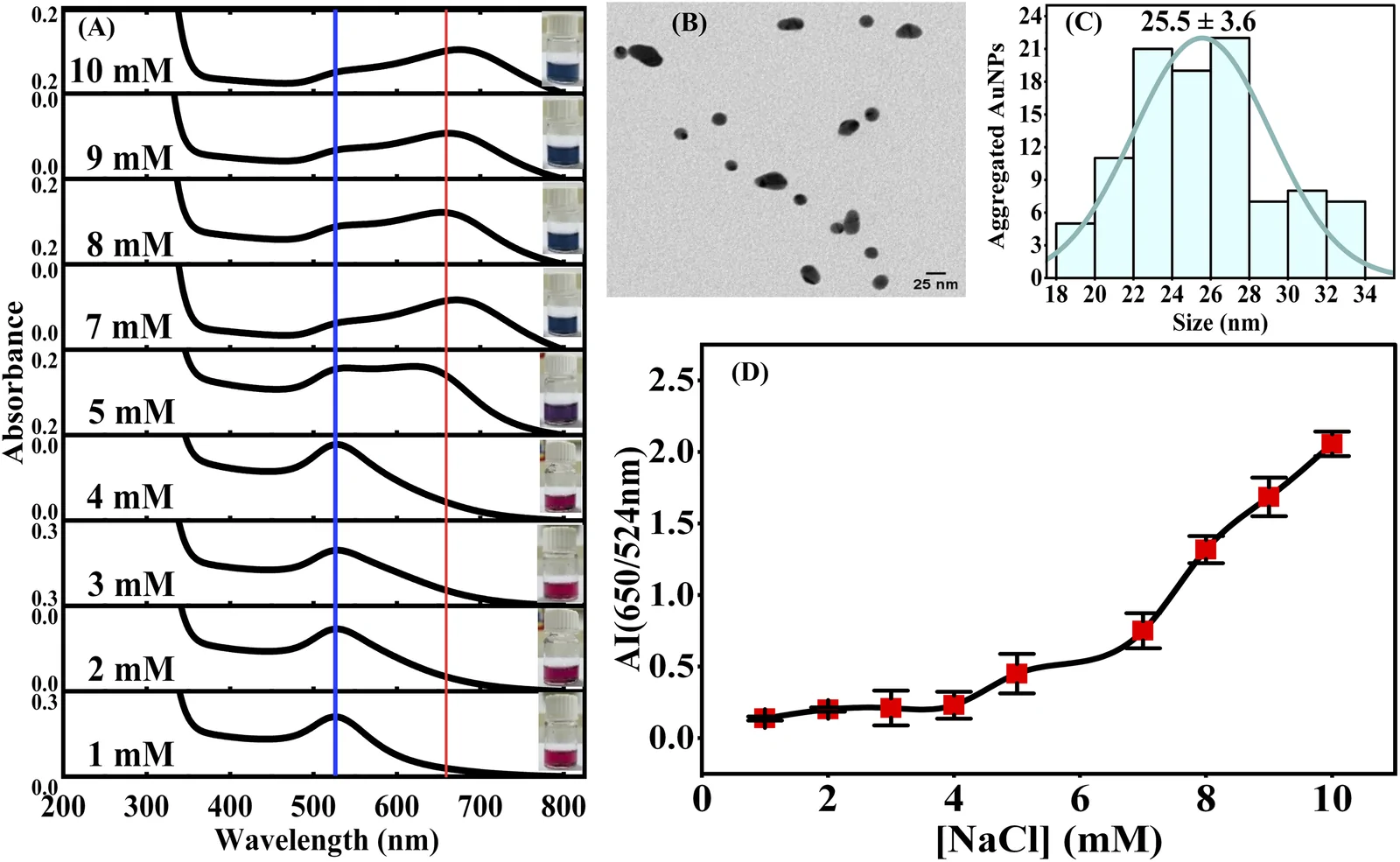
(A) UV–visible spectra of Au–4MBA nanoparticles recorded in the presence of increasing NaCl concentrations (1–10 mM). Insets show photographs of the corresponding dispersions. (B) Representative TEM micrograph of the Au–4MBA suspension after exposure to 5 mM NaCl. (C) Size distribution of aggregated Au-4MBA nanoparticles. (D) Dependence of the aggregation index (*A*_650_/*A*_524_)on NaCl concentration.

However, the TEM image demonstrated in (Fig. 2B) supports the UV–visible observations for the effect of salt concentration on the colloidal stability. Representative TEM images at 5 mM NaCl revealed the formation of nanoparticle clusters with no observable change in individual particle morphology, confirming that the spectral changes originate from interparticle assembly rather than particle dissolution or structural transformation. TEM analysis showed that the primary Au–4MBA nanoparticles retained comparable individual particle dimensions following exposure to 5 mM NaCl. However, pronounced interparticle association and cluster formation were observed (Fig. 2B). Analysis of the resulting clusters revealed an average apparent aggregate size of 25.5 ± 3.6 nm (Fig. 2C), larger than the average diameter of the isolated Au–4MBA nanoparticles (10.23 ± 0.62 nm, Fig. 1B). The corresponding aggregate-size distribution is presented in Fig. 2C, providing quantitative support for NaCl-induced aggregation. To quantitatively assess the influence of ionic strength on colloidal stability, the AI values was evaluated as a function of NaCl concentration (Fig. 2D). The AI remained relatively low between 1 and 4 mM NaCl, increasing only slightly from 0.14 to 0.23. This limited variation indicates that the Au–4MBA nanoparticles largely preserved their dispersed state within this concentration range, consistent with the negligible spectral broadening observed in the UV–visible spectra. A more pronounced increase was observed at 5 mM NaCl, where the AI nearly doubled to approximately 0.45, indicating the onset of significant interparticle association. Further increases in electrolyte concentration (7 to 10 mM NaCl) resulted in a sharp and continuous rise in AI respectively. Relative to the value obtained at 1 mM NaCl, the AI increased by nearly fifteen-fold at 10 mM NaCl, demonstrating extensive nanoparticle assembly under highly screened conditions. The concentration-dependent AI profile exhibits two distinct regimes. Below 5 mM NaCl, only minor changes in AI are observed, suggesting that electrostatic repulsion provided by the surface-bound 4-MBA ligands remains sufficient to maintain colloidal stability. At concentrations of 5 mM and above, the AI increases sharply, indicating the onset of extensive nanoparticle aggregation and plasmonic coupling. The abrupt change in slope near 5 mM NaCl therefore identifies a critical destabilization region, beyond which electrolyte screening effectively overcomes the repulsive barrier surrounding the nanoparticles. The simultaneous growth of the long-wavelength plasmon band and reduction of the primary LSPR intensity observed in Fig. 2A are fully consistent with this trend. The sigmoidal nature of the AI–concentration relationship further suggests that aggregation does not progress linearly with ionic strength. Instead, particle association accelerates once a sufficient degree of charge screening has been achieved, leading to cooperative growth of nanoparticle clusters. This behaviour is characteristic of electrolyte-induced destabilization in charged colloidal systems, where compression of the electrical double layer reduces the interparticle separation required for attractive van der Waals interactions to dominate. Consequently, the AI provides quantitative confirmation that 5 mM NaCl represents the threshold region for destabilization of the Au–4MBA colloidal suspension and was therefore selected for subsequent aggregation-suppression studies.

### 3.3. Dye-mediated suppression of aggregation

To investigate the influence of CR dye on electrolyte-induced colloidal destabilization, the CR dye was introduced into the Au–4MBA nanoparticle suspension containing 5 mM NaCl, where significant aggregation had previously been observed. The spectral evolution of the Au–4MBA colloidal system changed significantly in the presence of CR dye.

The UV–visible spectra in Fig. 3 eveal a clear distinction between the Au–4MBA suspension containing 5 mM NaCl and the corresponding system containing both NaCl and CR. The spectral band found in 600–700 nm region due to plasmonic coupling disappeared when the CR was present. The introduction of 10 µM CR remained the dominant spectral band found at near 524 nm for Au-4MBA nanoparticles, indicating that the dye effectively counteracts the destabilizing influence of the electrolyte. Meanwhile, the corresponding photographic images provide direct visual evidence of this stabilization process. By comparison, the Congo Red-containing system retained a wine-red appearance similar to that of the initially dispersed nanoparticles. The photographic image reveals that the absence of visible precipitation or pronounced colour evolution is consistent with the spectroscopic observations and suggests that large-scale aggregate formation is effectively inhibited. However, free CR exhibits characteristic electronic absorption bands at approximately 247 and 498 nm, whereas Au–4MBA nanoparticles display a plasmon resonance at 524 nm. The association of CR with the Au–4MBA/NaCl system, the spectral response is governed predominantly by the nanoparticle plasmon band rather than the intrinsic absorption of the dye. The preservation of the plasmon maximum together with suppression of the aggregation-associated absorption indicates that the observed response originates from modification of colloidal behaviour rather than simple spectral superposition. Finally, the presence of CR contributed to maintaining the primary plasmon band near 524 nm in a dispersed state while significantly suppressing the development of the aggregation-associated absorption feature. This behaviour is likely associated with modification of the Au–4MBA interfacial environment following dye adsorption.

**Fig. 3:**
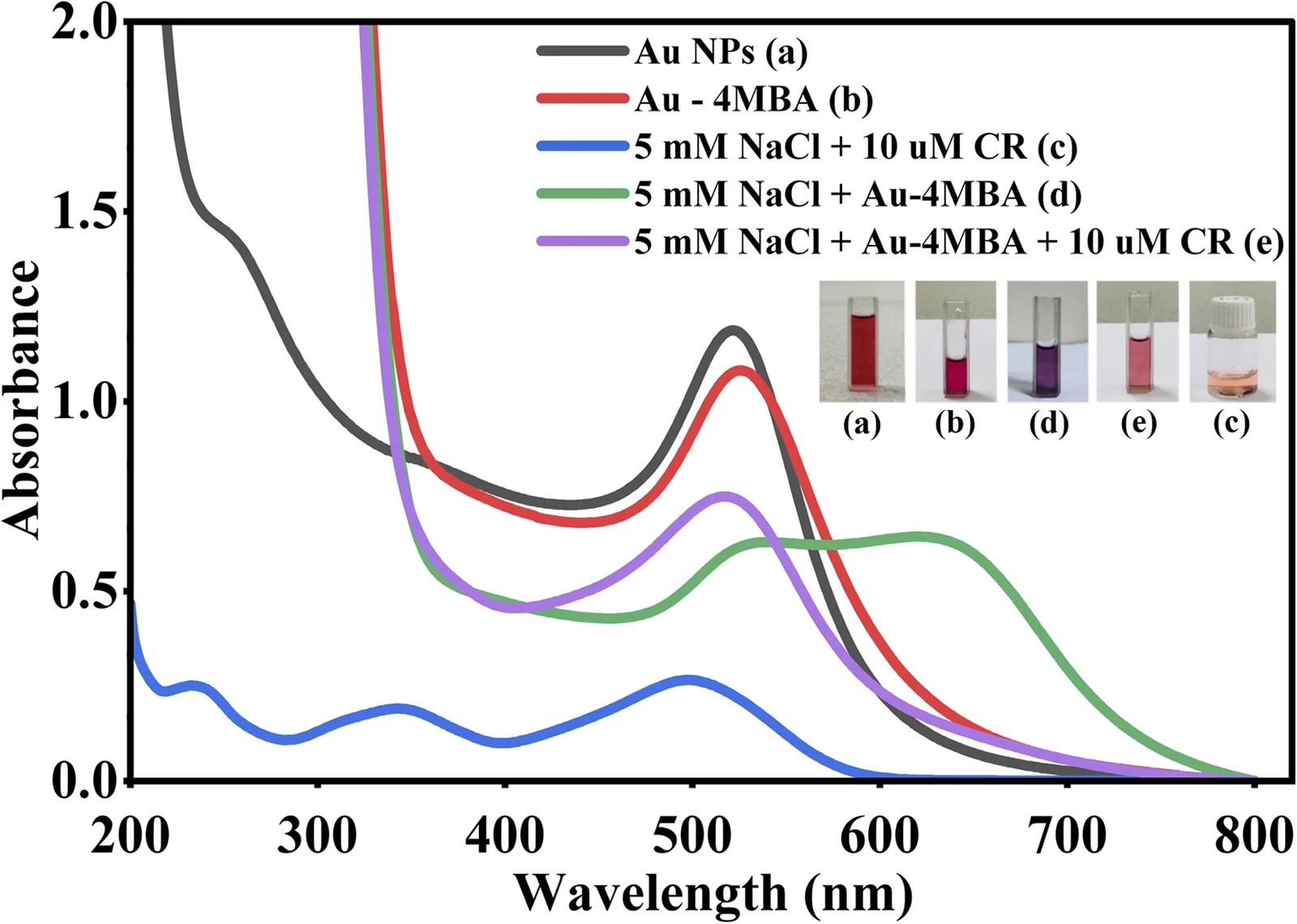
UV–visible spectra of citrate-stabilized Au nanoparticles (AuNPs), Au–4MBA nanoparticles, Au–4MBA nanoparticles in the presence of 5 mM NaCl, Congo Red (CR, 10 µM), and Au–4MBA nanoparticles containing 5 mM NaCl and 10 µM CR. Insets show photographic images of the corresponding colloidal suspensions.

### 3.4. Effect of dye concentration

The influence of CR concentration on the colloidal behavior of the Au–4MBA/5 mM NaCl system was investigated over the concentration range of 1–50 µM (Fig. 4). The UV–visible spectra (Fig. 4A) represents a well-defined LSPR band without the appearance of a distinct aggregation-associated absorption feature in the 650–700 nm region across the entire concentration. The corresponding photographic images likewise showed preservation of the characteristic red colour of the colloidal suspensions. These observations demonstrate that CR effectively suppresses the electrolyte-induced aggregation pathway under the investigated conditions.

**Fig. 4:**
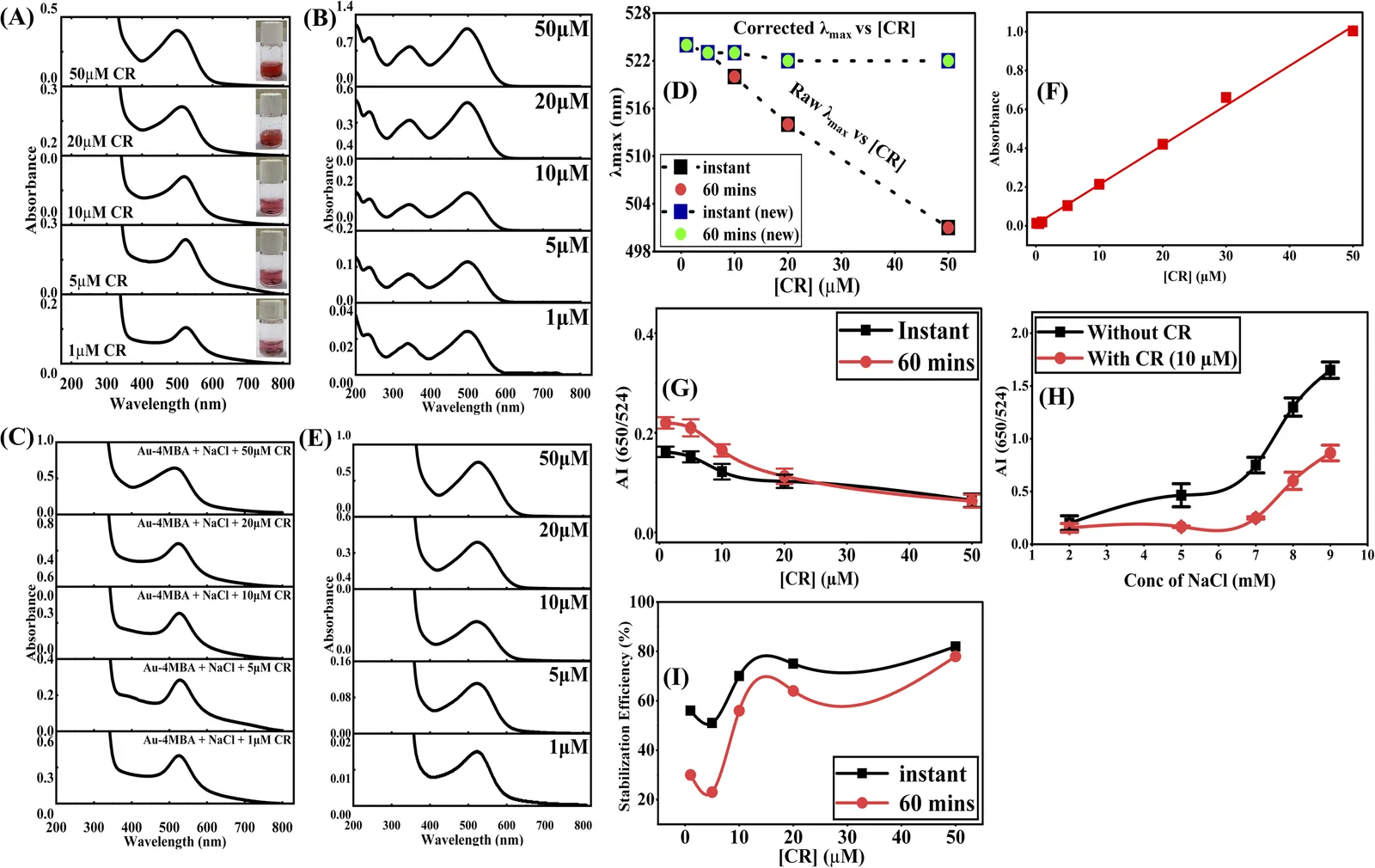
Effect of Congo Red (CR) concentration on the stabilization and optical response of Au–4MBA nanoparticles in the presence of NaCl. (A) Uncorrected UV–visible spectra of Au–4MBA nanoparticles containing 5 mM NaCl and different concentrations of CR (1–50 µM). (B) Concentration-matched UV–visible spectra of CR (1–50 µM) in the presence of 5 mM NaCl used as background controls. (C) CR-corrected UV–visible spectra of the Au–4MBA/5 mM NaCl/CR systems obtained by subtraction of the corresponding CR/NaCl control spectra. (D) Comparison of the apparent *λ*_max_ obtained from the uncorrected spectra and the nanoparticle-associated *λ*_max_ obtained following spectral correction as a function of CR concentration. (E) UV–visible absorption spectra of the supernatants obtained after separation of Au–4MBA nanoparticles from dispersions containing 5 mM NaCl and different initial CR concentrations: (a) 50, (b) 20, (c) 10, (d) 5, and (e) 1 µM. (F) Calibration curve of CR constructed under the corresponding electrolyte conditions. (G) Aggregation index (AI) as a function of CR concentration immediately after preparation and after 60 min. (H) Comparison of AI as a function of NaCl concentration in the absence and presence of 10 µM CR. (I) SE as a function of CR concentration immediately after preparation and after 60 min.

The uncorrected UV–visible spectra (Fig. 4A) initially appeared to exhibit a concentration-dependent displacement of the dominant absorption maximum toward shorter wavelengths. At CR concentrations of 1–10 µM, the apparent maximum remained close to the characteristic Au–4MBA LSPR at approximately 524 nm, whereas it shifted to approximately 520–521 nm at 20 µM CR and approached ∼501 nm at 50 µM CR. Comparable spectral positions were observed immediately after preparation and after 1 h, indicating that the observed spectral profiles remained relatively stable over this period. However, because CR itself exhibits a strong electronic absorption band near 500 nm, the apparent displacement of the spectral maximum at higher CR concentrations could not be attributed directly to a corresponding shift of the Au–4MBA LSPR.

To determine the contribution of intrinsic CR absorption, concentration-matched CR controls containing 5 mM NaCl were recorded under the same experimental conditions (Fig. 4B). The intensity of the characteristic CR absorption band near 500 nm increased markedly with CR concentration and became particularly prominent at the higher concentrations. This spectral feature substantially overlaps with the Au–4MBA LSPR region and therefore contributes increasingly to the composite absorption profile of the Au–4MBA/NaCl/CR system. Concentration-matched CR/5 mM NaCl spectra were subsequently subtracted from the corresponding Au–4MBA/5 mM NaCl/CR spectra to isolate the nanoparticle-associated plasmonic contribution (Fig. 4C). Following spectral correction, the Au–4MBA plasmon maximum remained within a relatively narrow wavelength range of approximately 522–524 nm throughout the investigated CR concentration range (Fig. 4D). In contrast, the apparent maximum obtained from the uncorrected spectra progressively shifted toward ∼501 nm at 50 µM CR. The substantial difference between the raw and corrected λ_max_ trends demonstrates that the pronounced apparent blue shift observed at high CR concentration arises predominantly from increasing spectral overlap with the intrinsic CR absorption band rather than from an equivalent displacement of the Au–4MBA LSPR. The corrected spectra therefore provide a more reliable representation of the plasmonic response of the nanoparticles in the presence of increasing CR concentrations. Meanwhile, the intrinsic CR absorption does not alter the principal concentration-dependent stabilization behavior of the Au–4MBA nanoparticles. The long-wavelength absorption associated with interparticle plasmon coupling in the 650–700 nm region progressively decreased as the CR concentration increased. Since the intrinsic electronic absorption of CR is predominantly centered near 500 nm and contributes only weakly within the 650–700 nm region, the progressive reduction of the aggregation-associated plasmonic response provides a more reliable spectroscopic measure of aggregation suppression. These observations indicate that CR restricts the close interparticle approach required for strong plasmon coupling, thereby maintaining Au–4MBA nanoparticles in a more dispersed state under electrolyte-rich conditions.

The quantitative analysis performed according to the method described in the section 2.7. The UV spectra of supernatant and calibration curve was demonstrated in Fig. 4E and F which was used for the quantitative analysis. The performance of quantitative analysis revealed CR association percentages of approximately 11, 16, 20, 15, and 26.39% at initial CR concentrations of 1, 5, 10, 20, and 50 µM, respectively. The fractional association varied across the investigated concentration range, while the absolute amount of nanoparticle-associated CR increased markedly from 0.11 µM at 1 µM CR to 13.20 µM at 50 µM CR. This increase indicates greater association of CR with the nanoparticle-containing fraction at higher dye concentrations and provides experimental support for the interaction of CR with the Au–4MBA system. The observed association behavior is consistent with the concentration-dependent suppression of the long-wavelength aggregation response. Association of CR with the 4-MBA-functionalized interface may introduce additional interfacial interactions and steric constraints that restrict the close approach of neighboring nanoparticles required for strong interparticle plasmon coupling. Consequently, the increased amount of nanoparticle-associated CR at higher concentrations is consistent with the improved resistance of the Au–4MBA dispersion to NaCl-induced aggregation.

The aggregation index further supported this interpretation. The concentration-dependent stabilization was further quantified using the AI (Fig. 4G). The AI decreased from approximately 0.18 ± 0.01 at 1 µM CR to 0.07 ± 0.01 at 50 µM CR immediately after preparation, corresponding to an overall reduction of approximately 61% across the investigated concentration range. A comparable trend was observed after 1 h, with the AI decreasing from approximately 0.22 to 0.07. The progressive decrease in AI is consistent with reduced interparticle plasmon coupling and demonstrates enhanced resistance of the Au–4MBA nanoparticles to NaCl-induced aggregation as the CR concentration increases. The protective effect of CR was also examined over a broader NaCl concentration range by comparing the aggregation behavior of Au–4MBA nanoparticles in the absence and presence of 10 µM CR (Fig. 4H). In the absence of CR, the AI increased markedly from approximately 0.20 ± 0.063 at 2 mM NaCl to 1.65 ± 0.077 at 9 mM NaCl, indicating extensive electrolyte-induced interparticle coupling. In contrast, lower AI values were observed over the same NaCl concentration range in the presence of 10 µM CR. At 5 mM NaCl, for example, the AI decreased from approximately 0.46 ± 0.1 in the absence of CR to approximately 0.16 ± 0.07 after CR addition, corresponding to a reduction of approximately 65%. Even at 9 mM NaCl, where pronounced aggregation occurred in the CR-free system, the AI remained substantially lower (∼0.87 ± 0.07) in the presence of CR. These results demonstrate that the protective effect of CR persists over a range of electrolyte concentrations and remains evident under conditions that otherwise promote substantial aggregation of Au–4MBA nanoparticles. The stabilization efficiency (SE) was further calculated from the aggregation indices measured in the presence and absence of CR (Fig. 4I). The stabilizing influence of CR was further quantified using the stabilization efficiency (SE), calculated according to:
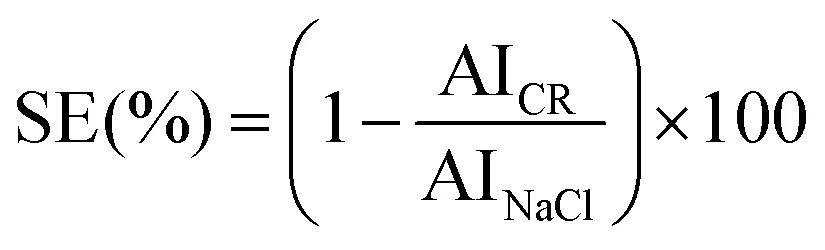
where AI_CR_ and AI_NaCl_ represent the aggregation indices measured in the presence and absence of CR, respectively. The stabilization efficiency increased overall from approximately 56% at 1 µM CR to approximately 82% at 50 µM CR immediately after preparation, with substantial stabilization observed at CR concentrations of 10 µM and above. These findings indicate that higher CR concentrations provide more effective protection against salt-induced aggregation. The AI and stabilization-efficiency analyses are fully consistent with the spectroscopic observations. Increasing CR concentration simultaneously reduces the extent of plasmonic coupling and improves colloidal stability while maintaining a dispersed nanoparticle population. The accompanying concentration-dependent blue shift of the LSPR band further reveals that CR does not merely prevent aggregation but also influences the local environment of the Au–4MBA interface.

### 3.5. Temperature-dependent aggregation behaviour

The temperature-dependent behaviour of the Au–4MBA colloidal system was examined between 10 and 50 °C under the critical destabilization condition of 5 mM NaCl to determine whether thermal energy influences nanoparticle assembly and whether CR retains its aggregation-suppressing capability over this temperature range (Fig. 5). In the absence of CR, the spectral response remained remarkably stable throughout the investigated interval (Fig. 5A). The plasmon maximum was observed at 524 nm at 10 °C and remained within 524 ± 1 nm up to 50 °C. The AI exhibited only limited variation with temperature, remaining within the range of 0.122–0.200 between 10 and 50 °C (Fig. 5C). This behaviour indicates that temperature exerts minimal influence on the aggregation state of the system. The absence of any systematic increase in AI suggests that elevated temperature does not accelerate nanoparticle association despite the increased Brownian motion expected at higher temperatures. Furthermore, the corresponding photographs show no detectable colour transition throughout the investigated range, indicating preservation of the colloidal state.

**Fig. 5:**
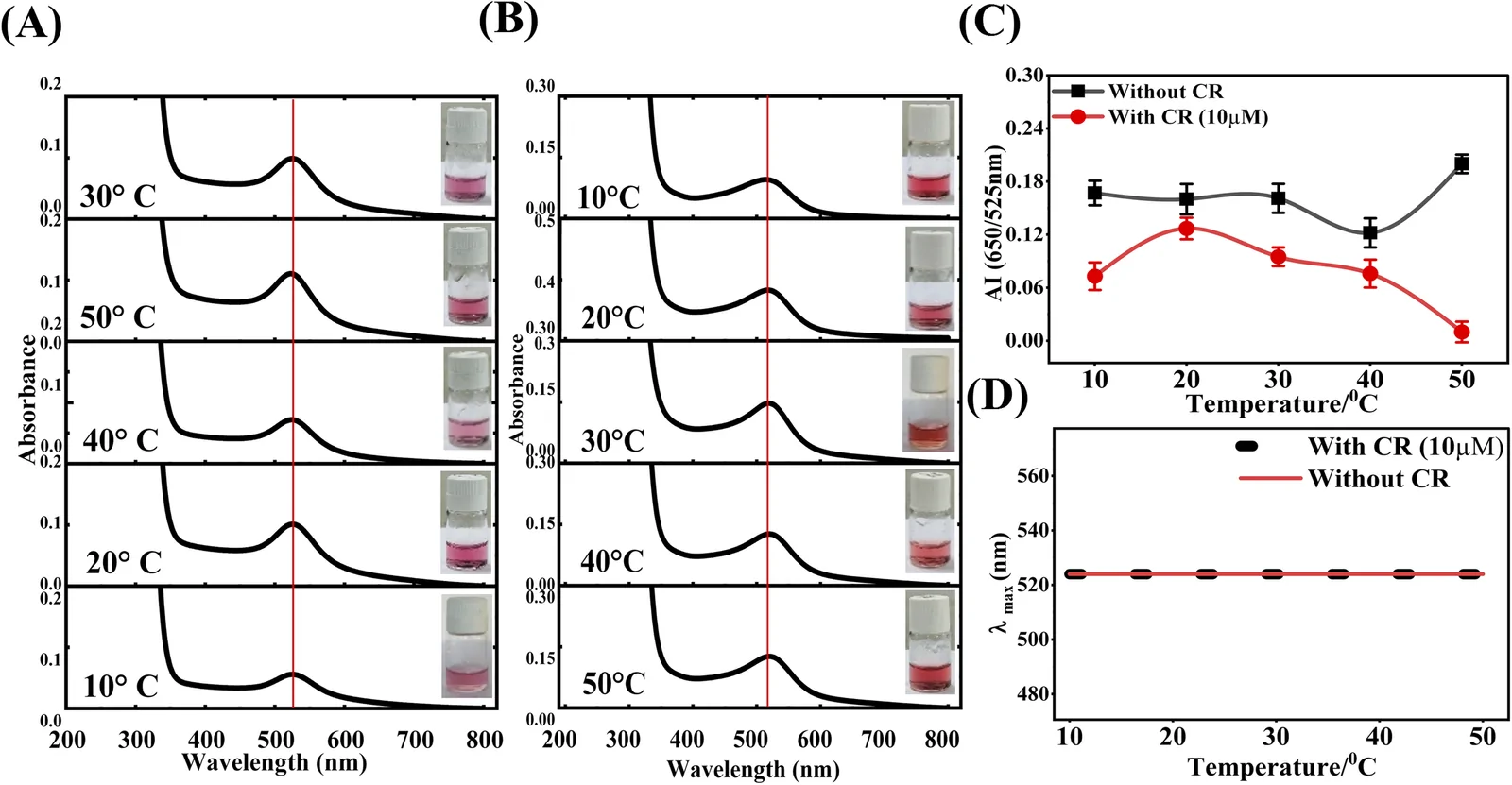
Effect of temperature on the colloidal stability of Au–4MBA nanoparticles under 5 mM NaCl conditions. (A) Temperature-dependent UV–visible spectra of Au–4MBA nanoparticles without Congo Red and corresponding photographs of the dispersions. (B) Temperature-dependent UV–visible spectra of Au–4MBA nanoparticles containing 10 µM Congo Red and corresponding photographs. (C) Aggregation index (AI = *A*_650_/*A*_524_) as a function of temperature for the dye-free and CR-containing systems. (D) Temperature dependence of the plasmon resonance wavelength (*λ*_max_).

A comparable temperature-independent behaviour was observed in the presence of 10 µM CR (Fig. 5B). The primary plasmon band remained near 524 nm across the entire temperature range, while the AI remained consistently low, varying between 0.010 and 0.126. These results indicate that temperature has little influence on the stability of the CR-containing system. The photographic images further support this observation, showing retention of the characteristic red colour of the dispersion at all temperatures. The *λ*_max_ profile shown in Fig. 5D provides additional evidence for the thermal robustness of the system. For both dye-free and CR-containing suspensions, the plasmon maximum remained constant within experimental uncertainty over the entire temperature range. The total variation in *λ*_max_ was less than 1 nm, indicating that temperature does not measurably alter the dielectric environment surrounding the nanoparticles or induce detectable changes in interparticle spacing. Such behavior contrasts with thermally sensitive colloidal systems, where aggregation is often accompanied by spectral broadening and significant wavelength shifts. More importantly, comparison of the AI profiles reveals that the stabilizing effect of CR greatly exceeds the influence of temperature. The dye-free system exhibited only limited variation in AI with temperature. In contrast, significantly lower AI values were observed in the presence of CR, demonstrating that the dye effectively suppresses aggregation over the entire temperature range investigated. Therefore, the magnitude of the stabilization effect is substantially greater than the magnitude of the thermal effect. This observation suggests that colloidal behavior is governed primarily by interfacial interactions associated with the Au–4MBA/CR system rather than by temperature-dependent changes in particle mobility. Mechanistically, increasing temperature would be expected to enhance nanoparticle diffusion and collision frequency. If the stabilizing interactions responsible for colloidal integrity were weak, such conditions would favor aggregation through increased particle–particle encounters. The absence of any corresponding increase in AI suggests that the interfacial barrier preventing nanoparticle association remains intact throughout the investigated temperature range. Consequently, the thermal energy available between 10 and 50 °C appears insufficient to overcome the stabilizing interactions operating at the Au–4MBA interface, either in the absence or presence of CR.

### 3.6. pH-dependent colloidal stability

The pH-dependent colloidal behaviour of Au–4MBA nanoparticles was investigated under 5 mM NaCl conditions to examine how variations in the ionization state of the surface-bound 4MBA ligands influence nanoparticle stability. A pronounced transition from an aggregated to a dispersed state was observed as the pH increased from 5 to 8–9, highlighting the critical role of surface-charge regulation in controlling interparticle interactions (Fig. 6). At pH 5, the UV–visible spectra exhibited secondary broad absorption band (∼670 nm) together with a substantial attenuation of the primary plasmon resonance at 524 nm when the CR was absent (Fig. 6A). The aggregation-associated band became more intense than the original plasmon band in the absence of CR. The corresponding colour transition from wine-red to blue-purple further confirms the formation of aggregated nanoparticle assemblies. Consistent with these observations, AI reached approximately 1.10 (Fig. 6D). Following the addition of 10 µM CR, the intensity of the long-wavelength plasmon band decreased noticeably and the AI was reduced to approximately 0.70, corresponding to nearly 36% suppression of aggregation. Although the extent of aggregation was reduced, the continued presence of the aggregation band together with the blue-purple colour confirms that nanoparticle association still occurs under acidic conditions. Upon increasing the pH to 8 and 9, the colloidal behaviour changed markedly (Fig. 6B and C). The characteristic plasmon resonance remained centred near 524 nm, while the aggregation-associated absorption feature disappeared almost completely. Simultaneously, the dispersions retained their characteristic wine-red appearance both in the absence and presence of CR, indicating preservation of the dispersed colloidal state. The AI decreased dramatically to approximately 0.10 and 0.05 at pH 8 for the dye-free and CR-containing systems, respectively, and remained close to these values at pH 9. Notably, the reduction in AI from approximately 1.10 at pH 5 to approximately 0.10 at pH 8 represents nearly a 90% decrease in aggregation intensity, identifying ligand deprotonation as the dominant factor governing colloidal stability under electrolyte conditions. The minimal difference between pH 8 and pH 9 further suggests that the major stability transition occurs between acidic and mildly alkaline conditions. The observed pH dependence can be rationalized by considering the ionization behaviour of the surface-bound 4-MBA ligands (Fig. 7). Under acidic conditions, protonation of the carboxylate groups converts negatively charged –COO^−^ functionalities into neutral –COOH species, substantially reducing the surface charge density of the nanoparticles. In the presence of 5 mM NaCl, where the electrical double layer is already partially compressed, this reduction in charge weakens electrostatic repulsion and facilitates close particle approach. Consequently, nanoparticles undergo interparticle association, producing strong plasmonic coupling and the emergence of the broad absorption band near 670 nm. At pH 8 and 9, the 4-MBA shell remains predominantly deprotonated, maintaining a high density of negatively charged carboxylate groups on the nanoparticle surface. The resulting electrostatic repulsion effectively counterbalances electrolyte-induced double-layer compression and preserves sufficient interparticle separation to prevent aggregation. As a result, the characteristic plasmon resonance at 524 nm is retained, AI values remain low, and no visible colour transition occurs. The role of CR is strongly dependent on the charge state of the underlying ligand shell. As illustrated schematically in Fig. 7, CR provides an additional stabilizing contribution through adsorption at the nanoparticle interface, introducing supplementary electrostatic and steric barriers that oppose particle association. This contribution becomes particularly evident at pH 5, where the aggregation index decreases from approximately 1.10 to 0.70 following dye addition. However, the stabilizing influence of CR cannot fully compensate for the substantial loss of electrostatic repulsion resulting from ligand protonation. Under alkaline conditions, where the deprotonated 4MBA layer already provides strong electrostatic stabilization, CR functions primarily as an auxiliary stabilizing component, producing only modest additional reductions in AI.

**Fig. 6:**
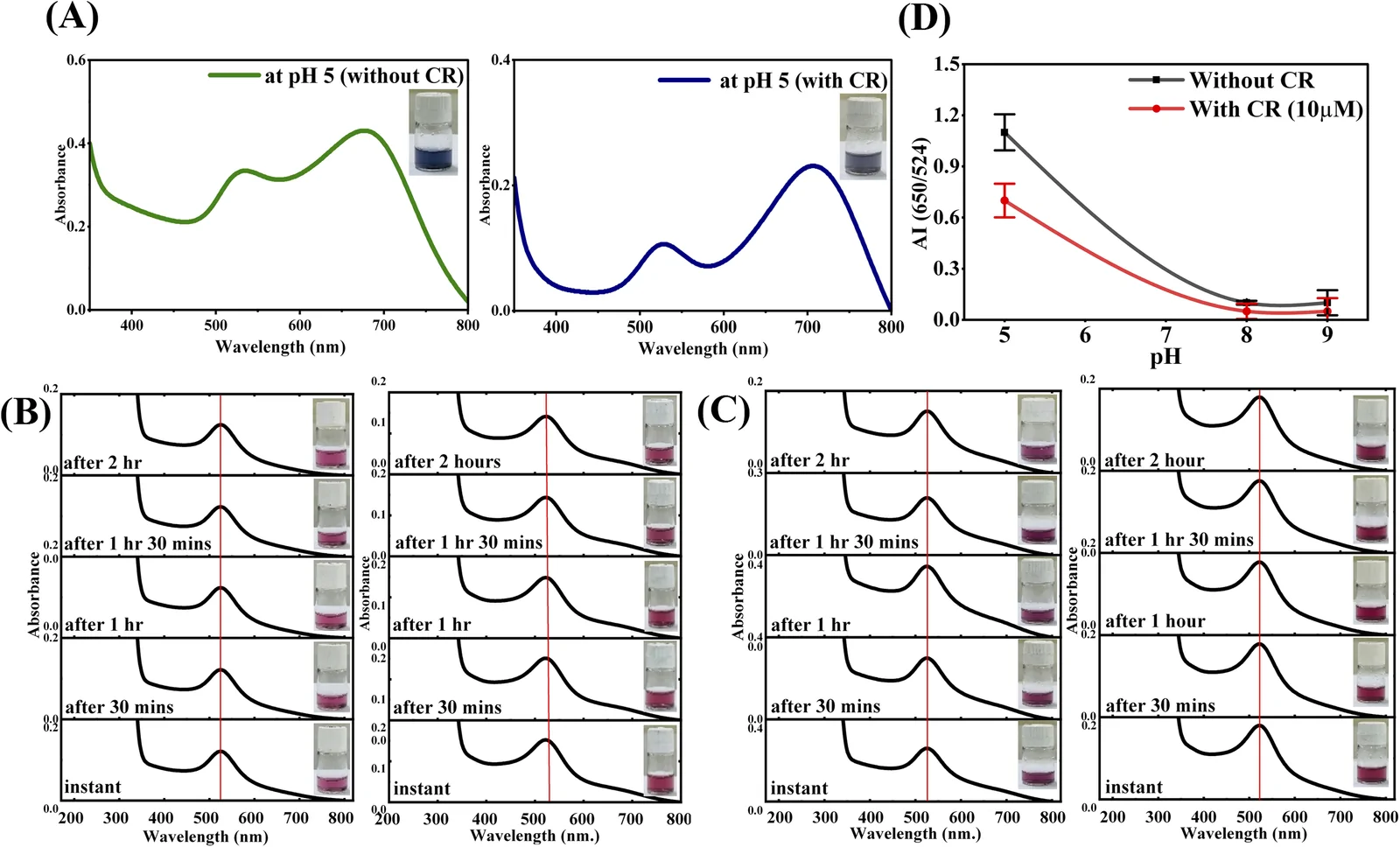
Effect of pH on the colloidal stability of Au–4MBA nanoparticles in the presence of 5 mM NaCl. (A) UV–visible spectra and corresponding photographs of Au–4MBA nanoparticles at pH 5 in the absence and presence of 10 µM Congo Red (CR). (B) UV–visible spectra and photographs of the corresponding systems at pH 8. (C) UV–visible spectra and photographs at pH 9. (D) Aggregation index (AI = *A*_650_/*A*_524_) as a function of pH for Au–4MBA nanoparticles with and without 10 µM CR.

**Fig. 7:**
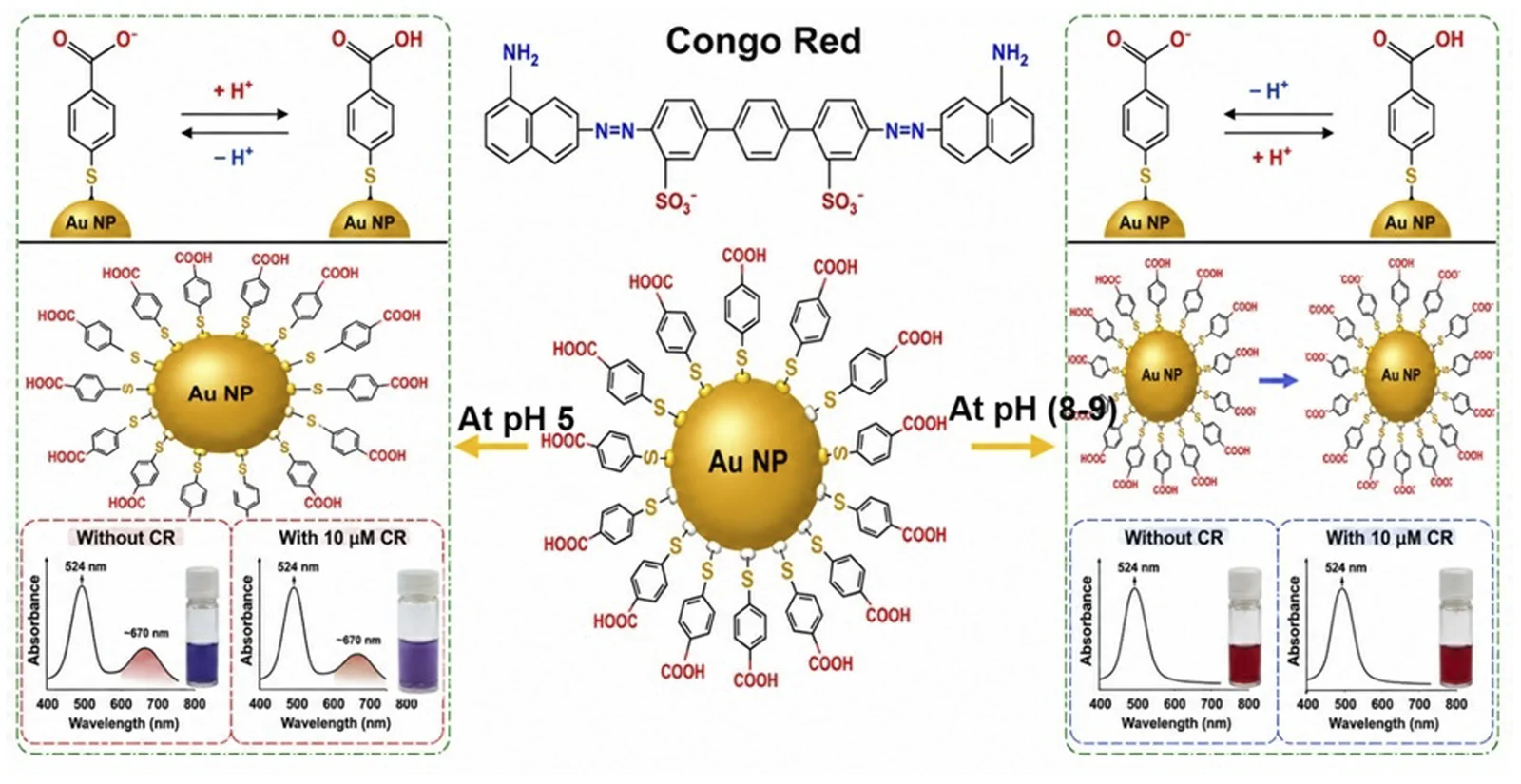
Schematic illustration of the pH-dependent colloidal behaviour of Au–4MBA nanoparticles in the presence of 5 mM NaCl. pH investigation is demonstrated in presence and absence of CR dye.

### 3.7. Time-dependent stability of Au–4MBA nanoparticles

The temporal evolution of the Au–4MBA colloidal system was investigated to establish the intrinsic stability of the ligand-functionalized nanoparticles and to examine how electrolyte addition and CR influence colloidal stability and aggregation kinetics. The combined spectroscopic and quantitative analyses reveal three distinct stability regimes: long-term stability of Au–4MBA nanoparticles in the absence of electrolyte, progressive aggregation following NaCl addition, and substantial suppression of aggregation in the presence of CR. As shown in Fig. 8A, Au–4MBA nanoparticles remained highly stable during 720 min of storage without added electrolyte. The UV–visible spectra exhibited negligible variation throughout the monitoring period, with the LSPR band remaining centered at 524 ± 1 nm and no detectable absorption appearing in the long-wavelength region. The absence of spectral broadening or aggregation-associated plasmon coupling demonstrates that the surface-bound 4-MBA layer effectively prevents spontaneous nanoparticle association. These observations confirm the excellent intrinsic colloidal stability of the functionalized nanoparticles and suggest that the Au–S linkage and ligand shell remain intact over prolonged storage.

**Fig. 8:**
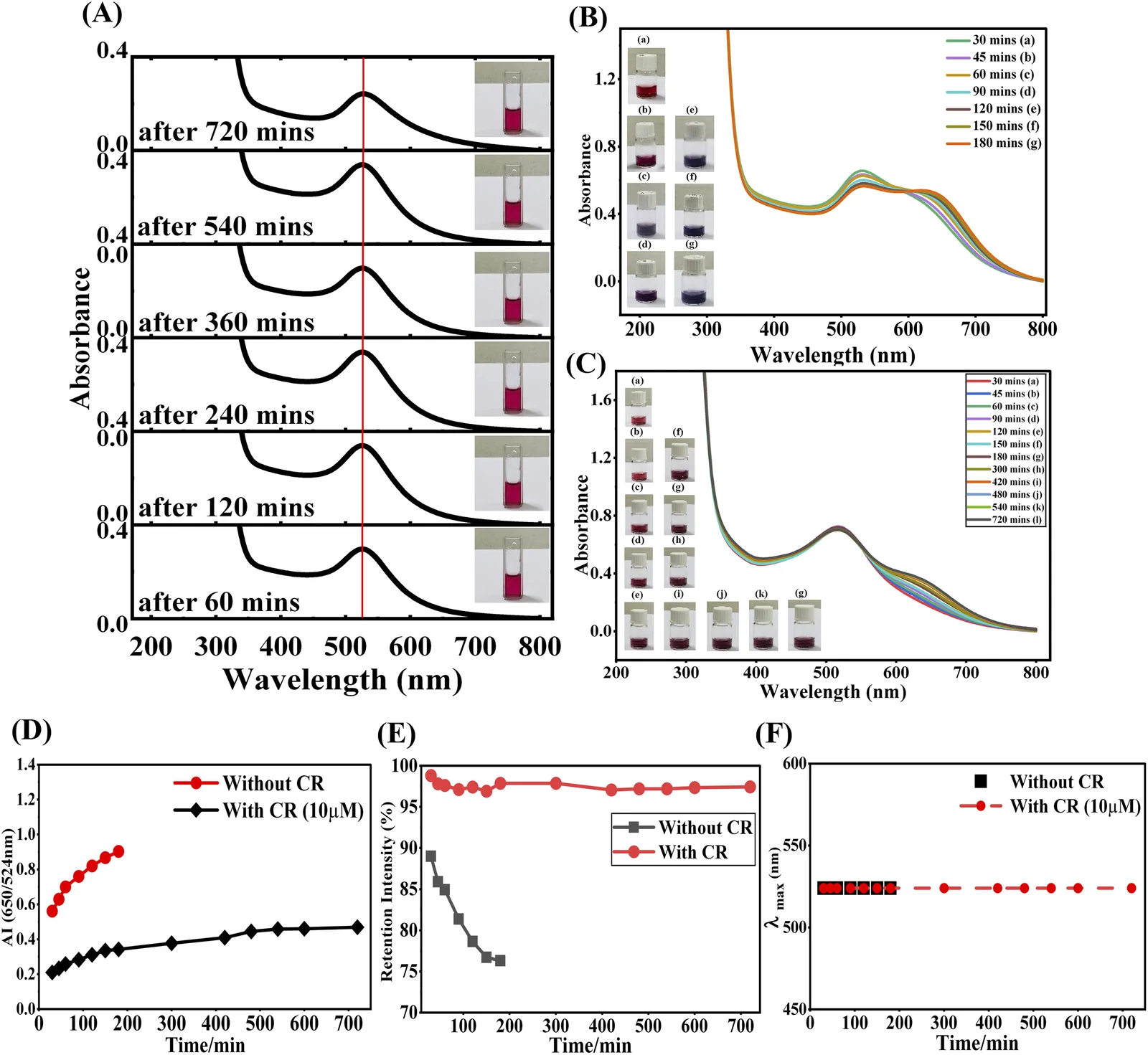
Time-dependent stability of Au–4MBA nanoparticles under different colloidal conditions. (A) UV–visible spectra of Au–4MBA nanoparticles monitored over seven days in the absence of added electrolyte. (B) Temporal spectral evolution of Au–4MBA nanoparticles in the presence of 5 mM NaCl recorded between 30 and 180 min. (C) Temporal spectral evolution of Au–4MBA nanoparticles in the presence of 5 mM NaCl and 10 µM CR recorded between 30 and 720 min. (D) Aggregation index (AI = *A*_650_/*A*_524_) as a function of incubation time for Au–4MBA + 5 mM NaCl and Au–4MBA + 5 mM NaCl + 10 µM CR. (E) Retention of the plasmon-band intensity at 524 nm (*A*_524_/*A*_524_, 0 × 100) as a function of time for the corresponding systems. (F) Variation of the localized surface plasmon resonance wavelength (*λ*_max_) with incubation time.

The temporal behavior changed markedly after introducing 5 mM NaCl (Fig. 8B). Between 30 and 180 min, the intensity of the primary plasmon band progressively decreased, accompanied by continuous growth of absorption in the 620–700 nm region. The development of this broad long-wavelength feature is characteristic of plasmonic coupling between neighboring nanoparticles and reflects the gradual formation of aggregated assemblies. This spectral evolution is quantitatively supported by the AI, which increased from approximately 0.56 at 30 min to 0.90 after 180 min (Fig. 8D), corresponding to an increase of nearly 61%. Simultaneously, the retention of the 524 nm plasmon intensity decreased from approximately 89% to 76% (Fig. 8E), confirming progressive loss of the dispersed nanoparticle population. Despite these intensity changes, the plasmon maximum remained essentially unchanged throughout the monitoring period (Fig. 8F), suggesting that destabilization proceeds predominantly through interparticle association rather than particle growth or irreversible precipitation. The addition of 10 µM CR to the Au–4MBA/5 mM NaCl dispersion, the aggregation pathway was markedly altered (Fig. 8C). The spectra recorded over 12 h retained a well-defined plasmon band near 524 nm and exhibited only limited growth of long-wavelength absorption. Consistent with this behavior, the AI increased gradually from approximately 0.21 at 30 min to 0.47 after 720 min (Fig. 8D), remaining substantially lower than that observed for the NaCl-only system. Even after 12 h, the AI remained approximately 48% lower than the value measured for the NaCl-treated dispersion after only 180 min. This suppression of aggregation is further reflected in the retention profile, where the CR-containing system maintained 97–99% of its initial plasmon intensity throughout the entire monitoring period (Fig. 8E). Moreover, *λ*_max_ remained constant at 524 ± 1 nm over the investigated time range (Fig. 8F), confirming preservation of the plasmonic characteristics of the dispersion. This observed differences in aggregation behavior can be attributed to changes in the nanoparticle interfacial environment. Following NaCl addition, compression of the electrical double layer may progressively weakens electrostatic repulsion between Au–4MBA nanoparticles, allowing attractive interparticle interactions to dominate and resulting in time-dependent plasmonic coupling. Adsorption of CR at the nanoparticle interface may introduces an additional stabilizing barrier that hinders close particle approach. The extended aromatic framework and multiple sulfonate functionalities of CR likely contribute to stabilization through a combination of interfacial adsorption, steric protection, and electrostatic effects. Consequently, the formation of plasmonically coupled nanoparticle assemblies is substantially reduce, resulting in lower aggregation indices, higher plasmon-intensity retention, and slower destabilization kinetics.

### 3.8. Spectral characteristics of the dyes and their response to electrolyte

To investigate how molecular structure influences the stability of Au–4MBA nanoparticles under electrolyte conditions, a series of structurally distinct dyes, including ARS, CR, EB, MO, and MB were selected for comparative evaluation. The optical characteristics of these dyes were first examined under the experimental conditions employed in this study to establish a reference for subsequent nanoparticle–dye interaction studies (Fig. 9A). The individual dyes exhibited distinct absorption profiles arising from their respective chromophoric structures. ARS displayed a strong absorption band near 265 nm together with a broader visible feature centered around 500 nm whereas EB showed a comparatively sharp absorption band at approximately 530 nm. MO and MB presented characteristic absorption maxima near 465 and 665 nm, respectively. The corresponding photographs highlight the distinct colours associated with each dye and reflect the differences in their electronic structures.

**Fig. 9:**
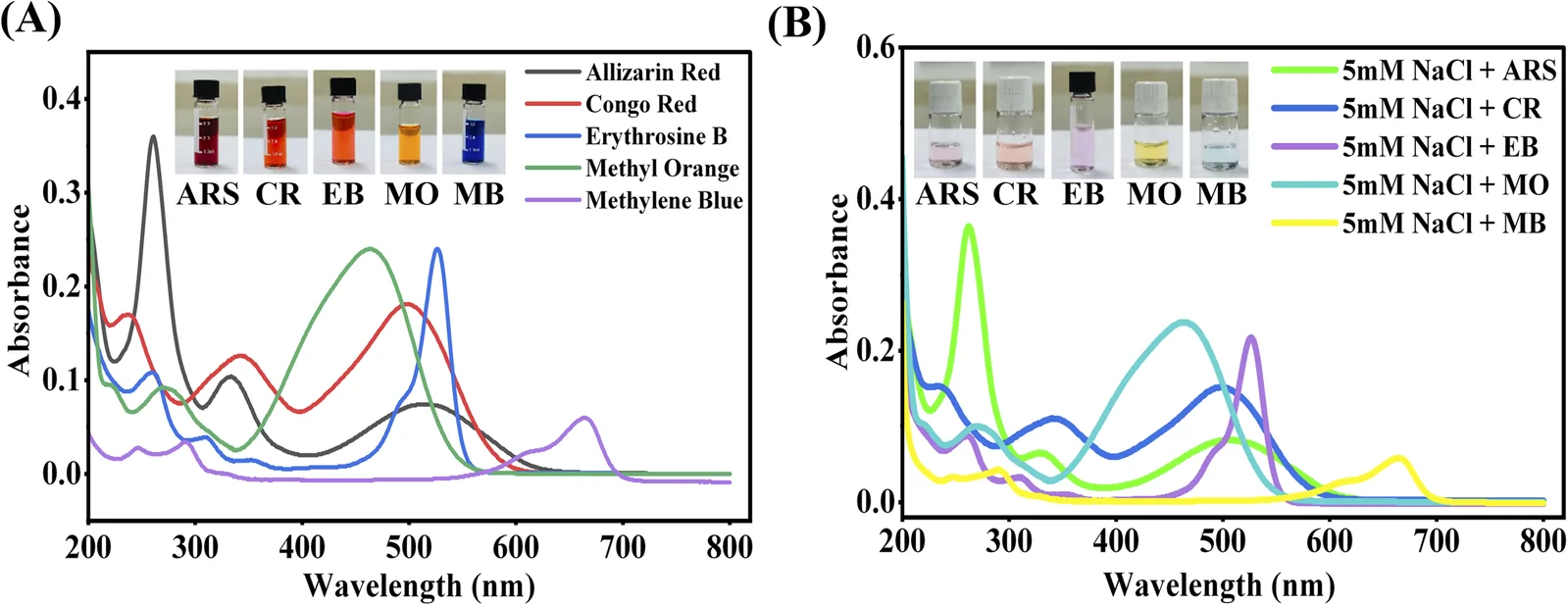
Spectral characteristics of the investigated dyes and their response to the presence of 5 mM NaCl. (A) UV–visible absorption spectra and corresponding photographs of Alizarin Red S (ARS), Congo Red (CR), Erythrosine B (EB), Methyl Orange (MO), and Methylene Blue (MB) in aqueous solution. (B) UV–visible absorption spectra and corresponding photographs of the same dyes in the presence of 5 mM NaCl. The preservation of the characteristic absorption features after electrolyte addition demonstrates that the dyes remain molecularly dispersed under the experimental conditions employed in the Au–4MBA aggregation studies.

The distinct spectral profiles reflect variations in molecular architecture, charge distribution, degree of π-conjugation, and functional-group composition among the investigated dyes. These structural features are expected to influence the strength and mode of adsorption at the Au–4MBA interface and may therefore contribute to differences in their ability to modulate nanoparticle aggregation and colloidal stability under electrolyte conditions. The spectral behaviour of the dyes was subsequently evaluated in the presence of 5 mM NaCl, corresponding to the critical electrolyte concentration employed throughout the nanoparticle aggregation studies. As shown in Fig. 9B, the characteristic absorption bands of all dyes remained clearly identifiable after electrolyte addition.

### 3.9. Comparative analysis of different dyes

To determine whether the aggregation-suppression behaviour observed for CR is unique to a specific molecular structure or represents a more general phenomenon, a series of structurally diverse dyes, namely ARS, CR, EB, MO, and MB, were evaluated under identical experimental conditions. The selected dyes differ substantially in molecular dimensions, aromatic surface area, charge distribution, degree of π-conjugation, and functional-group composition, providing a suitable platform for examining how molecular architecture influences the stability of Au–4MBA nanoparticles under electrolyte conditions. The individual dyes exhibit distinct absorption profiles within the visible region (Fig. 9), which can contribute differently to the composite spectra of the corresponding Au–4MBA/NaCl/dye systems. To distinguish the intrinsic dye contribution from the nanoparticle response, the spectra were evaluated after correction using the corresponding dye controls. Differences in the nanoparticle-associated spectral features remained after correction, indicating that the observed variation among the dye systems cannot be attributed solely to their intrinsic optical absorption. The corrected UV–visible spectra obtained in the presence of 5 and 7 mM NaCl revealed clear differences in the response of the Au–4MBA nanoparticles to the investigated dyes under electrolyte conditions (Fig. 10A and B).

**Fig. 10:**
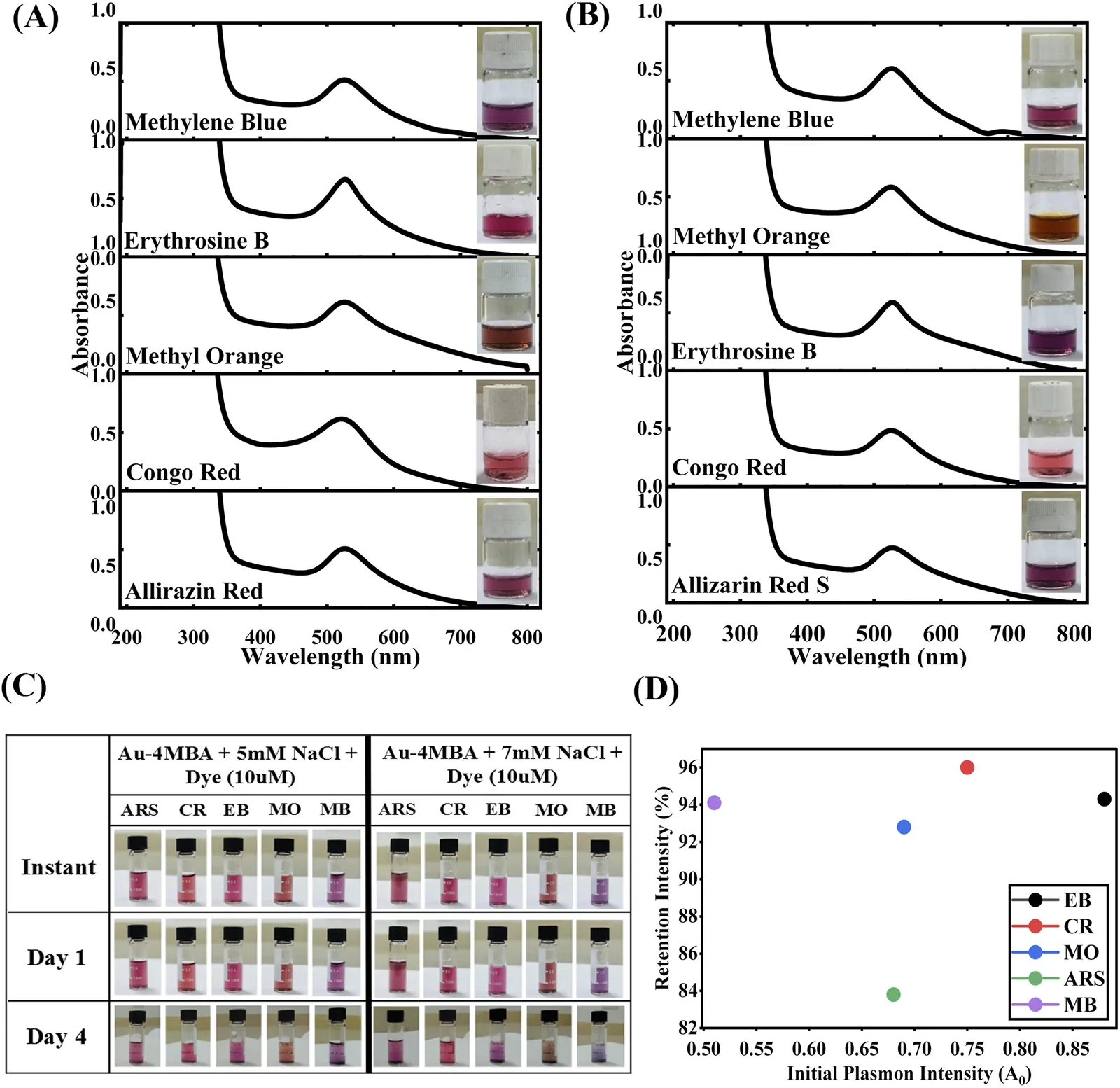
Comparative evaluation of the stabilization behaviour of structurally distinct dyes toward Au–4MBA nanoparticles under electrolyte conditions. (A) UV–visible spectra of Au–4MBA nanoparticles containing different dyes in the presence of 5 mM NaCl. (B) UV–visible spectra of the corresponding systems in the presence of 7 mM NaCl. (C) Photographic images showing the colloidal stability of the dye-containing Au–4MBA dispersions immediately after preparation and after 1 and 4 days of storage. (D) Correlation between the initial plasmon intensity (*A*_0_) and the corresponding intensity retention after 1 h, providing a comparative assessment of dye-mediated stabilization performance.

In all cases, the primary plasmon band remained centered near 524–530 nm, indicating that complete electrolyte-induced aggregation was suppressed. Nevertheless, notable variations in plasmon intensity and long-wavelength absorption were observed among the dyes. EB and CR consistently produced the strongest plasmon responses at both electrolyte concentrations, whereas MO and ARS exhibited intermediate behaviour. MB generated the weakest plasmon signal and displayed the most pronounced long-wavelength absorption, indicating comparatively lower resistance to electrolyte-induced destabilization. Increasing the NaCl concentration from 5 to 7 mM produced only modest changes in the overall spectral profiles, and the relative performance of the dyes remained largely unchanged, suggesting that the stabilizing influence of each dye persists under increased ionic strength. The photographic stability study further supported the spectroscopic observations (Fig. 10C). Immediately after preparation and after one day of storage, all dye-containing dispersions remained visually homogeneous with no obvious evidence of extensive aggregation. More distinct differences became apparent after four days. Suspensions containing CR and EB retained a relatively uniform appearance with minimal sediment formation, consistent with their strong plasmon responses and limited long-wavelength absorption. In contrast, the ARS containing system exhibited a noticeable shift toward a bluish-purple appearance, particularly at 7 mM NaCl, suggesting progressive nanoparticle association during storage. MO showed moderate sediment accumulation, whereas MB displayed the most pronounced sedimentation among the investigated dyes. The close correspondence between the spectroscopic and photographic observations indicates that the stability trends identified from the UV–visible measurements are maintained during storage. A quantitative comparison of the dye-dependent stabilization behaviour is presented in Fig. 10D, where the initial plasmon intensity is plotted against the corresponding intensity retention after 1 h. CR and EB occupy the upper-right region of the plot, reflecting a favourable combination of strong plasmon response and high intensity retention. MO exhibits intermediate performance, whereas ARS shows a more pronounced decline in plasmon intensity over time. Although MB displays relatively high intensity retention, its substantially lower initial plasmon intensity distinguishes it from the more effective stabilizing dyes. These observations demonstrate that intensity retention alone does not fully describe stabilization performance and highlight the importance of considering both plasmon preservation and temporal stability when evaluating dye-mediated stabilization. The observed differences in stabilization behavior appear to be associated with the structural characteristics of the investigated dyes. Dyes possessing larger aromatic frameworks and multiple charged functionalities, such as CR and EB, exhibited superior resistance to electrolyte-induced aggregation. In contrast, ARS and MO provided less effective stabilization, which may be associated with differences in their molecular dimensions and interfacial interactions. The weaker performance of MB suggests that molecular charge alone does not govern colloidal stability. The results suggest that differences in molecular size, aromatic surface area, and functional-group distribution may influence interfacial association and, consequently, the ability of the dyes to suppress nanoparticle aggregation.^31,32^ A schematic representation of the proposed interactions between the dyes and the Au–4MBA interface is presented in Fig. 11. The stabilization trend cannot be explained solely by electrostatic effects. If charge were the dominant factor, the anionic dyes would be expected to exhibit comparable behaviour. Instead, the observed differences suggest that molecular dimensions, aromatic characteristics, and functional-group arrangement may contribute to the different stabilization behaviors of the investigated dyes.^33,34^ The aromatic nature of the 4-MBA ligand shell further suggests that π–π interactions, hydrogen bonding, and electrostatic interactions may contribute to dye association with the functionalized nanoparticle interface; however, the specific molecular interactions and adsorption configurations were not directly determined in the present study.^35,36^ Taken together, the spectroscopic, photographic, and quantitative analyses consistently demonstrate that CR and EB provide the most effective stabilization of the Au–4MBA system, followed by MO and ARS, whereas MB exhibits the weakest stabilizing effect. Accordingly, the overall stabilization trend may be expressed as CR ≈ EB > MO > ARS >> MB. These findings are consistent with a structure-dependent influence on the colloidal stability of Au–4MBA nanoparticles under electrolyte conditions.

**Fig. 11:**
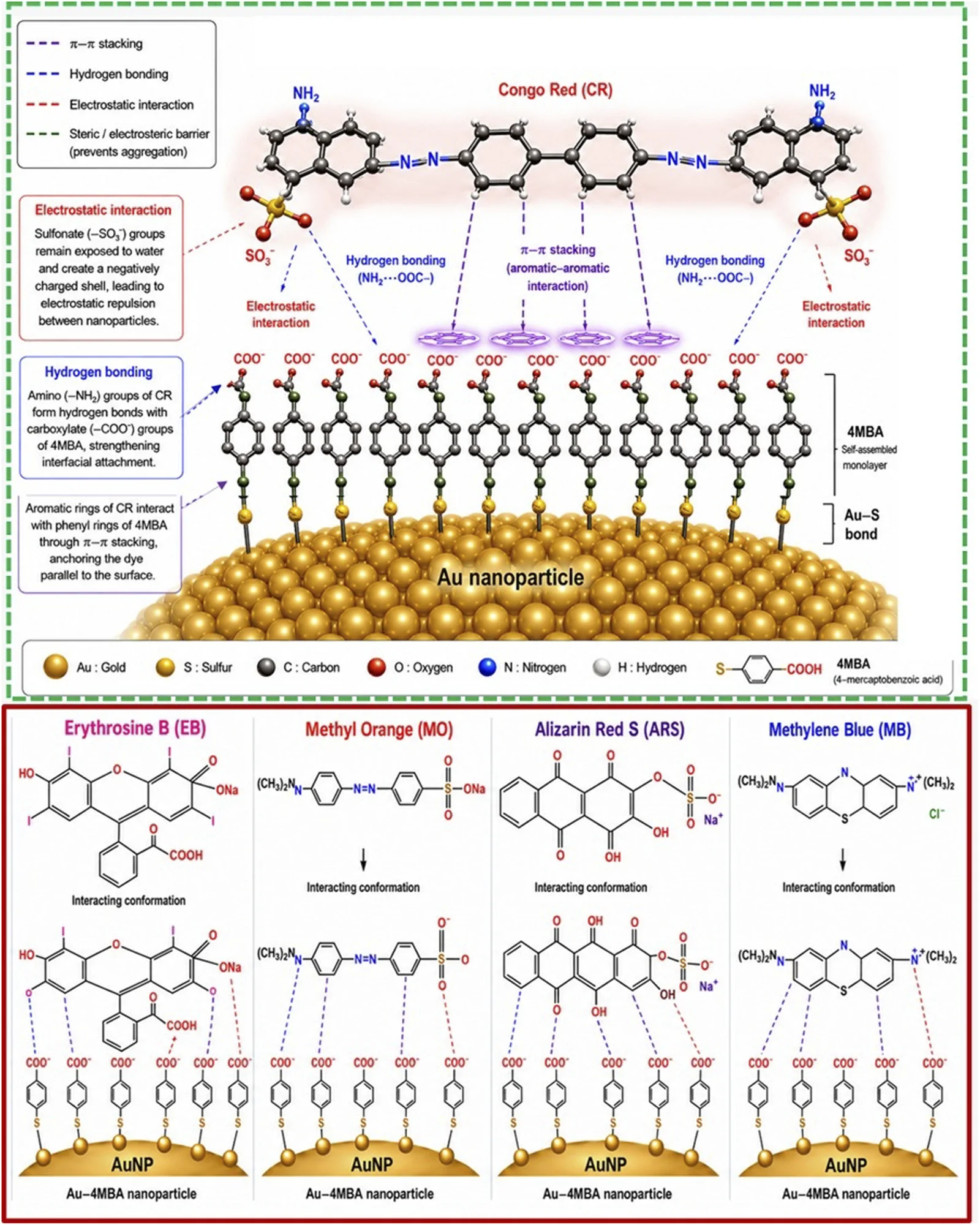
Schematic representation of the proposed interactions potentially contributing to the stabilization of Au–4MBA nanoparticles by CR, EB, MO, ARS, and MB.

## 4. Conclusions

In this study, the aggregation behaviour of Au–4MBA nanoparticles was systematically investigated under electrolyte conditions, and the influence of aromatic dyes on colloidal stability was elucidated. The colloidal response of the Au–4MBA system was highly sensitive to electrolyte-induced destabilization. However, the presence of Congo Red substantially altered the observed aggregation behavior and promoted retention of the dispersed plasmonic state. The concentration dependent stabilization behaviour further highlights the importance of molecular-level interfacial organization in governing nanoparticle assembly. Systematic investigations revealed that colloidal stability was strongly dependent on dye concentration and pH but largely insensitive to temperature within the investigated range. The pronounced pH dependence emphasized the critical role of the ionization state of the 4MBA ligand shell in regulating interparticle interactions and colloidal behaviour. Comparative evaluation of structurally distinct dyes revealed differences in stabilization behavior that appear to be associated with their molecular characteristics. Collectively, the experimental observations are consistent with a proposed adsorption-mediated electrosteric stabilization mechanism, in which association of the dye molecules with the Au–4MBA interface may restrict close particle approach and reduce interparticle plasmon coupling. Beyond identifying an effective stabilization strategy, this work provides broader insight into how interfacial molecular structure governs the behaviour of ligand-functionalized plasmonic nanomaterials and offers an outline for improving colloidal stability in electrolyte-rich environments relevant to sensing and related applications.

## Conflicts of interest

There is no conflict of interest exists declared by the authors.

## Data Availability

The data supporting the findings of this study are available within the article. Additional data are available from the corresponding author upon reasonable request.
